# The Effect of Point-of-Care Testing at Triage: An Observational Study in a Teaching Hospital in Saudi Arabia

**DOI:** 10.5811/westjem.2018.6.38217

**Published:** 2018-07-26

**Authors:** Jameel Abualenain, Ahd Almarzouki, Rawan Saimaldaher, Mark S. Zocchi, Jesse M. Pines

**Affiliations:** * King Abdulaziz University, Department of Emergency Medicine, Jeddah, Saudi Arabia; † King Abdulaziz University Hospital, Department of Emergency Medicine, Jeddah, Saudi Arabia; ‡ George Washington University, Department of Emergency Medicine, Washington, District of Columbia; § George Washington University, Center for Healthcare Innovation & Policy Research, Washington, District of Columbia

## Abstract

**Introduction:**

Prolonged waiting times during episodes of emergency department (ED) crowding are associated with poor outcomes. Point-of-care testing (POCT) at ED triage prior to physician evaluation may help identify critically ill patients. We studied the impact of ED POCT in a single ED with a high degree of crowding for patients with high-risk complaints who were triaged as non-critically ill.

**Methods:**

We conducted the study from April–July 2017 at King Abdulaziz University (KAU) Hospital in Jeddah, Saudi Arabia. Patients with one of seven complaints received triage POCT. The primary outcome was whether POCT results at triage resulted in immediate transfer of the patient from the waiting room into the ED. Secondary outcomes were whether the triage nurse felt that the POCT results were useful, and whether triage POCT changed triage acuity. We used simple descriptive statistics to summarize the data.

**Results:**

A total of 94 patients were enrolled and received i-STAT® POCT. The most common symptoms and triage protocols were for chest pain (42%), abdominal pain (31%), and shortness of breath (22%). In 11 cases (12%), care was changed as a result of triage POCT. In 12 cases (13%), triage level was changed. The triage nurse found POCT helpful in 93% of cases.

**Conclusion:**

In this ED, triage POCT was a helpful adjunct at ED triage and resulted in immediate care (transfer to an ED room) in one in eight cases. Therefore, POCT at triage may be a useful adjunct to improve patient safety, particularly in crowded EDs.

## INTRODUCTION

Emergency departments (ED) worldwide are facing increasing crowding and prolonged waiting times, which are associated with poor outcomes.^1^–^5^ During episodes of ED crowding, patients experience critical care delays, increased errors, and commonly a poorer experience.^6^ Crowding can worsen outcomes for acute myocardial infarction, sepsis, and trauma.^7^,^8^ One area of particular concern is the ED waiting room, where patients with undifferentiated conditions wait before physician evaluation. Patients who are obviously critically ill rarely spend any time waiting. Yet some patients triaged as safe to wait actually have occult, severe conditions requiring immediate treatment, which this may go unrecognized in a crowded ED.

One intervention to help detect patients who require immediate care is point-of-care testing (POCT) at ED triage. Triage POCT occurs prior to physician evaluation and can aid in identifying clinically important and abnormal test results, reduce the time to detect critical illness, and identify patients safe to wait.^9^ In previous studies, we developed POCT triage protocols and found they changed management decisions in a simulation with triage nurses.^10^,^11^ We conducted a prospective study at a single, academic ED in Jeddah, Saudi Arabia, with critical crowding and long ED wait times for patients with high-risk complaints triaged as safe to wait. Our goal was to determine the effect of POCT on decisions to transfer patients from the waiting room to the main ED, changes to triage prioritization, and perceived utility of POCT by triage nurses.

## METHODS

### Study Design

We conducted a prospective, observational study of an ED POCT triage program. This was approved by the institutional review boards at George Washington University and King Abdulaziz University (KAU) Hospital in Jeddah, Saudi Arabia.

### Setting

This was an observational pilot study with a total enrollment of 94 patients. KAU is a tertiary care, academic medical center with 71 ED beds, 845 hospital beds, and an annual ED census of 66,000 visits. In April 2017 POCT became available, and enrollment took place from April to July 2017. Prior to POCT implementation, blood-based ED POCT was unavailable and no POCT was conducted at triage except for finger-stick glucose.

In Saudi Arabia the government has a very clear and strict law, which states that any patient presenting to any ED with a life- or limb-threatening condition is to be attended to immediately, regardless of any other factors used to determine eligibility. However, other less-urgent or non-emergent conditions vary dramatically in terms of acceptance in EDs based on each facility’s regulations. Even governmental institutions vary dramatically in their eligibility criteria, which makes ED populations vary significantly based on an institution’s rules.

All patients who arrive to the ED are triaged using the Canadian Triage and Acuity Scale (CTAS), a five-level triage system corresponding to 1 (Resuscitation), 2 (Emergent), 3 (Urgent), 4 (Less Urgent), and 5 (Non-Urgent). During registration, patients are assigned in the health information system into their respective ED section (i.e., fast track or main ED) by their CTAS level (1–5). All CTAS 1–2 patients are registered with no restrictions and regardless of nationality. However, CTAS 3–5 patients are registered based on certain eligibility criteria. Eligible patients are citizens of Saudi Arabia/Gulf region and university/hospital staff including their dependents, regardless of nationality. Additionally, also eligible for registration are non-Saudis who are sponsored by individuals (not companies), some infectious diseases (assigned by the Ministry of Health), active cancer patients on chemotherapy, and some nationalities facing crisis in their homeland (decided by the government).

**Population Health Research Capsule**

What do we already know about this issue?

Point-of-care testing (POCT) at emergency department (ED) triage can aid in identifying clinically important and abnormal test results, reduce time to detect critical illness, and identify patients safe to wait.

What was the research question?

Does POCT affect decision to transfer patients from waiting room to main ED and change triage prioritization?

What was the major finding of the study?

Triage POCT directly resulted in immediate transfer of patients to ED room in one in eight cases. Triage level was changed in 13% of cases.

How does this improve population health?

POCT at triage is a helpful adjunct at ED triage and resulted in immediate care changes. It may be a useful adjunct to improve patient safety for waiting patients, particularly in crowded EDs.

Ineligible patients (CTAS 3–5 patients) are non-Saudis who hold health insurance through their sponsors (companies, agencies, etc.). Ineligible patients do not get registered and are advised to seek medical care in the private sector or governmental hospitals that offer services for the privately insured. In triage, all patients regardless of eligibility may receive electrocardiograms (ECGs); however, other testing is not done. Patients are seen by a physician after arrival in an ED room, where they have laboratory tests drawn and sent to a central laboratory adjacent to the ED.

### Subjects

The study sample consisted of registered patients triaged CTAS 2–5 with one of seven predefined conditions who received POCT at triage. Patients were enrolled in the study when the study team of nurses and medical interns was available. Potential study subjects meeting inclusion criteria were approached by an enroller. After consent, a nurse drew a venous blood sample and conducted POCT using i-STAT® (Abbott Point of Care, Inc., Princeton, NJ) based on a clinical protocol (Table 1).

i-STAT® POCT results are typically available in under 10 minutes. Test results were presented to the ED attending physician who determined whether the patient required immediate care. We used a structured data form to collect demographic and clinical information on each patient. The triage nurse was then asked to complete a brief survey on whether POCT was useful in determining urgency, and how it changed triage for the patient. This was done for every patient (See Appendix).

### Study Outcomes

The primary study outcome was whether the POCT results resulted in immediate transfer from the waiting room to the main ED. Secondary outcomes were whether the triage nurse felt that the POCT results were useful, and whether triage POCT changed the patient’s triage acuity.

### Data Analysis

We used simple descriptive statistics and completed analyses using Stata version 14.2 (StataCorp, College Station, TX).

## RESULTS

A total of 94 patients were enrolled. Average age was 59, and 48% were female. The triage protocols used the most were chest pain (42%), abdominal pain (31%), and shortness of breath (22%). CTAS 3 was the most common triage level (72%). The most common POCT was troponin (56%), followed by lactate (18%) (Table 2).

In 11 cases (12%), patients were moved directly from the waiting room to an ED room. In 12 cases (13%), the triage level was changed. While the triage nurse found POCT helpful in 93% of cases, triage POCT increased the level of concern in 12 cases (13%) and decreased it in six cases (6%). With respect to disposition, 30 patients (32%) left the ED before being seen by a physician (Table 3).

## DISCUSSION

We found that triage POCT was a helpful adjunct to triage in a crowded ED where patients experience long waits and commonly leave the ED before physician evaluation, even with potentially emergent conditions. Triage POCT directly resulted in immediate transfer of the patient to an ED room in one in eight cases. In addition, triage level was changed in several cases.

POCT was particularly helpful in diagnosing atypical presentations of acute myocardial infraction. For example, a mid-30s male patient with several cardiovascular comorbidities and atypical chest pain of longstanding duration yet well appearing and in no acute distress had a positive troponin of 0.89 and was immediately brought to an ED room and diagnosed with non-ST segment elevation myocardial infarction. In another case, a mid-40s male presented with vague generalized weakness and was prioritized as CTAS 3. POCT showed a positive troponin (0.55) and a low pH (6.8). An immediate ECG showed an ST-segment acute myocardial infarction and the patient was transferred to the cardiac catheterization laboratory. In a third case, a mid-50s male presented with fatigue and generalized weakness and had been in the waiting room most of the day. After enrollment, he was found to have a low pH (7.1), a low bicarbonate (13), and hyperkalemia (6.7 meq/L). He was also brought back for immediate care. POCT troponin was the test that most changed triage decisions. It helped to change six (30%) of the 18 cases. These six patients had normal ECGs and negative POCT troponin.

Several prior studies have also explored how POCT changes triage decisions.^8^,^9^ One study implemented triage POCT for similar conditions (i.e., chest pain, infection, and older adults) and found that triage POCT resulted in immediate transfer in 6% of cases, lower than in our study.^8^ Differences in rates are likely due to differences in the ED environment, and the policies surrounding triage prioritization. Specifically at KAU, considerably higher risk patients must commonly wait and may not receive treatment. This approach is more common outside of the United States, particularly in countries such as Saudi Arabia, the Gulf region, India, and others.^1^ Therefore, the value of POCT at triage may be magnified in those settings.

## LIMITATIONS

There are several study limitations. This was a single-center study and may not generalize to other settings. It was a convenience sample of patients who presented during times when study staff were available. Enrolling patients who presented at other times may have yielded different results. POCT can incur significant costs to implement and maintain. There was no follow-up done for patients who left without being seen. The research protocol stated that lactate should be tested in suspected sepsis cases, but during the study period no sepsis cases were enrolled. Triage decisions are by their nature subjective, and results may differ with different triage nurses or other local protocols. However, triage nurses at our hospital have a minimum of five years of ED experience, and all of them went through CTAS triage training conducted by emergency physicians (EP). Moreover, triage nurses have direct communication with all EPs on duty using mobile phones provided by the hospital, and they call frequently for any inquiries or concerns.

## CONCLUSION

POCT was a useful adjunct at triage and resulted in changes in triage level and immediate transfer to an ED room in one in eight cases, suggesting that point-of-care testing may improve patient safety in similar settings.

## Supplementary Information



## Figures and Tables

**Table 1:** Triage point-of-care protocol.

Condition	Inclusion	Exclusion	i-STAT POCT ordered
Symptoms of	≥ 40 yo OR cardiovascular risk factor OR congestive heart failure	-Chest trauma -Suspected respiratory infection -History of asthma OR COPD with bilateral wheezingNone	Troponin
chest or epigastric pain / shortness of breathSymptoms of generalized weakness	≥ 55 yo OR multiple comorbidity	None	Troponin CHEM8+ Lactate Hgb/Hct
Symptoms of abdominal pain (young female)	< 55 yo	Post-menopausal (medical and surgical menopause included)	Pregnancy test
Symptoms of abdominal pain (older pain)	≥ 55 yo OR multiple co-morbidity	None	Lactate
History of syncope (older patient)	≥ 40 yo OR multiple co-morbidity	None	Troponin Glucose
History of missed dialysis	None	None	CHEM8+
History of GI bleeding symptoms	None	Suspected benign etiology	Hgb/Hct
Suspected sepsis	≥ 2 SIRS criteria or debilitated/ill-appearing	≥ 18 years old	Lactate WBC

*POCT*, Point-of-care testing; *GI*, gastrointestinal; *Hgb/Hct*, hemoglobin/hematocrit; *SIRS*, systemic inflammatory response syndrome (temp >38°C (100.4°F) or < 36°C (96.8°F), heart rate > 90, respiratory rate > 20 or PaCO_2_ < 32 mm Hg, white blood count (WBC) > 12,000/mm^3^, < 4,000/mm^3^, or > 10% bands).

**Table 2:** Study subjects who received point-of-care testing at emergency department triage (n=94).

	No.	%
Female	45	47.9
Mean age (SD), years	58.8	(14.0)
Chief complaint		
Abdominal pain	29	30.9
Chest pain	39	41.5
Shortness of breath	21	22.3
Fatigue/weakness	6	6.4
Nausea/vomiting	4	4.3
Syncope	3	3.2
Triage level		
2	19	20.2
3	67	71.3
4	8	8.5
Disposition		
Admitted	18	28.1
Discharged	46	71.9
Left without being seen	30	31.9
POC test type		
Troponin only	53	56.4
Lactate only	17	18.1
BHCG only	11	11.7
Troponin, CHEM8+, Lactate, and HGB/HCT	6	6.4
CHEM8+ and PT/INR	4	4.3
Troponin and glucose	3	3.2

*SD*, standard deviation; *BHCG*, beta human chorionic gonadotropin; *POC*, point-of-care; *HGB*, hemoglobin; *HCT*, hematocrit; *PT*, prothrombin time; *INR*, international normalized ratio.

**Table 3:** Care management / triage survey results (n=94).

	No.	%
Total cases	94	100
Changed triage		
No change	82	87.2
CTAS 2-->3	5	5.3
CTAS 3-->2	7	7.4
Changed care		
Brought immediately to the main ED	11	11.7
POCT helpful	87	92.6

*CTAS*, Canadian Triage Acuity Scale; *ED*, emergency department; *POCT*, point-of-care testing.
