# Supplementary material for: The Effect of Point-of-Care Testing at Triage: An Observational Study in a Teaching Hospital in Saudi Arabia
Source: West J Emerg Med. 2018 Jul 26;19(5):884–8. doi: 10.5811/westjem.2018.6.38217 (PMC6123100; doi:10.5811/westjem.2018.6.38217)
Supplement: Supplementary file 1 [file wjem-19-884-s001.pdf]

Appendix: Triage Nurse Survey

STICKER

<u>TRIAGE NURSE SURVEY</u>

This survey should be administered to the triage nurse AFTER showing the Point-of-care laboratory results.

This patient was enrolled in a research study. You have seen the point-of-care (POC) laboratory results.

The triage level that you assigned was:	__________ (Study nurse to fill out)
Do knowing the POC lab results change the level of concern for the immediacy for which the patient should be seen?	☐ YES ☐ NO
If the answer is YES, was your level of concern:	☐ INCREASED – patient is *more* ill than your initial triage impression<br>☐ DECREASED – patient is *less* ill than your initial triage impression
What would your triage level have been had you known the POC lab results?	__________ (1 to 5)
Is your care of this patient changed by knowing the POC lab results?	☐ YES ☐ NO
If the answer is YES, how was it changed?	☐ Brought patient to the main ED immediately<br>☐ Administered intravenous fluids<br>☐ Administered aspirin<br>☐ Other ______________________
Was access to POC laboratory results helpful in the triage of this patient?	☐ YES ☐ NO
